# Marine Subsurface Microbial Community Shifts Across a Hydrothermal Gradient in Okinawa Trough Sediments

**DOI:** 10.1155/2016/2690329

**Published:** 2016-12-20

**Authors:** Leah D. Brandt, Christopher H. House

**Affiliations:** Department of Geosciences, The Pennsylvania State University, 220 Deike Building, University Park, PA 16802, USA

## Abstract

Sediments within the Okinawa back-arc basin overlay a subsurface hydrothermal network, creating intense temperature gradients with sediment depth and potential limits for microbial diversity. We investigated taxonomic changes across 45 m of recovered core with a temperature gradient of 3°C/m from the dynamic Iheya North Hydrothermal System. The interval transitions sharply from low-temperature marine mud to hydrothermally altered clay at 10 meters below seafloor (mbsf). Here, we present taxonomic results from an analysis of the 16S rRNA gene that support a conceptual model in which common marine subsurface taxa persist into the subsurface, while high temperature adapted archaeal taxa show localized peaks in abundances in the hydrothermal clay horizons. Specifically, the bacterial phylum Chloroflexi accounts for a major proportion of the total microbial community within the upper 10 mbsf, whereas high temperature archaea (Terrestrial Hot Spring Crenarchaeotic Group and methanotrophic archaea) appear in varying local abundances in deeper, hydrothermal clay horizons with higher* in situ* temperatures (up to 55°C, 15 mbsf). In addition, geochemical evidence suggests that methanotrophy may be occurring in various horizons. There is also relict DNA (i.e., DNA preserved after cell death) that persists in horizons where the conditions suitable for microbial communities have ceased.

## 1. Introduction

The marine subsurface hosts a diverse ecosystem of microbial life that has direct consequences on whether organic carbon or other elements are sequestered over geologic time or are recycled as active elements back into the ocean-atmosphere system [2]. The limits of life that define the microbial extent within marine subsurface sediments have remained unresolved; however, the increased temperature associated with sediment burial is often perceived as one of the major constraints [2]. There is evidence of microbial populations existing and thriving around hydrothermal vent emissions (reaching temperatures approaching 400°C) [3], but many recent studies have begun to focus on exploring whether such a hyperthermophilic biosphere exists at higher temperatures within the sedimentary subsurface (i.e., [4]). Rather than exploring the temperature limits at 4 km below seafloor, where temperatures are predicted by the geothermal gradient to approach 100°C, subsurface hydrothermal sediments have become ideal study sites because of their large thermal gradients over a much shorter vertical profile (e.g., Guaymas Basin, Juan de Fuca Ridge, Middle Valley, and Okinawa Trough). Deep sea sediments exist at predominantly low temperatures (~1–5°C); however, areas of new ocean crust formation (e.g., mid-ocean ridges) or zones of back-arc spreading create localized hydrothermal vent systems, emissions of high temperature fluids emanating from the subsurface as a result of magmatic degassing and subsurface water-rock reactions under high temperatures and pressures [5]. Fluids migrating through these sediments undergo heating and water-sediment interactions that create distinct subsurface geochemical conditions from cold marine sediments.

Back-arc basins are the result of the rifting away of a magmatic arc from a continental margin and can have significant sediment input from continental runoff, surface productivity, and/or the volcaniclastic debris [6]. In the case of the Okinawa back-arc basin system, extension/rifting occurring of the overriding plate coincides with its continental shelf. This continental margin-like geographic setting, being the transition from continental to oceanic crust, overlaying a subsurface hydrothermal system makes the Okinawa back-arc basin a unique marine environment from other sediment-hosted hydrothermal systems. Sediment profiles within this system are subject to intense temperature and alteration gradients, making it an ideal system to examine how the sedimentary biosphere may be affected through such gradients.

The Integrated Ocean Drilling Program (IODP) Expedition 331 recovered sediments within the Iheya North Hydrothermal Field in the Okinawa back-arc basin to explore the extent and diversity of the “subvent” biosphere. Site C0014, located 450 m away from the main hydrothermal mound (Figure S1 showing the spatial extent of Site C0014 within Iheya North Hydrothermal Field in Supplementary Material available online at http://dx.doi.org/10.1155/2016/2690329), was investigated to test for a taxonomically diverse microbial community across a temperature gradient increasing with depth. The sediment profile at Site C0014 exhibits a transition from hemipelagic ooze with pumiceous volcaniclastic sediments to a hydrothermally altered sequence of clays within the top ~10 mbsf of sediment [7]. Temperature measurements indicate a gradient of approximately 3°C/m (Table S1 and Figure S2(E)) [7], which is roughly an order of magnitude greater than continental margin sites (e.g., Cascadia Margin, IODP 311, and Costa Rica Margin, IODP 344) but is more gradual than intense, centimeter-scale gradients from other hot, surface sediments. Expanding upon studies that suggest the presence of microbial life deep into sediments [4, 8], this study is intended to provide a comprehensive analysis of the microbial community composition through a temperature gradient. In order to investigate the relationship between the microbial communities and the degree of increasing hydrothermal conditions in the Okinawa back-arc basin, we used high throughput sequencing of the 16S rRNA gene to produce a taxonomic analysis of the bacterial and archaeal communities down core at Site C0014 [9]. We use this study as a proxy of the distribution of life at the biotic fringe in deeper subsurface sediments, where we hypothesize that either (1) mesophilic taxa reach a threshold and are replaced by a higher temperature adapted community in deeper, hotter horizons or (2) there is minimal species change down to some horizon with no establishment of a (hyper) thermophilic community due to the dynamic nature of this subsurface hydrothermal system. Here, we report on the taxonomic changes through a temperature and geochemical gradient and speculate on the extent of the biosphere based on DNA recovery and geochemical measurements.

## 2. Experimental Procedures

### 2.1. Sample Collection and Extraction

All samples in this study were collected on IODP Expedition 331 at Sites C0014 and C0015. Sediment sections from Holes B, D, and G, cored within approximately 10 m of one another, were used in this study. Aliquots of sediment from all subcores were, in a sterile sampling environment, taken from the center of whole rounds stored at −80°C in sterile containers. Samples were shipped on dry ice to the Pennsylvania State University and were kept at −80°C until analysis. See Table S1 for a list of samples used in this study. Sample depths are reported in units of depth below seafloor, which only takes into account the distance of sampling section from the sediment-water interface and does not take into consideration consolidation of sediment, sediment composition, biostratigraphy, sediment age, or* in situ* temperature. The values reported in this study are the averages of the top and bottom depths of the subcore (see Table S1 for depth intervals of each core). DNA was extracted in quadruplicate for a total of 1-2 g sediment using the PowerSoil® DNA Isolation Kit (MoBio Laboratories, Inc.) with modifications as follows: step 1: 200 *μ*L sterile TE buffer was added to 0.25–0.5 sediment g in the PowerBead Tubes in addition to the C1 solution; step 5: PowerBead Tubes were homogenized for 30 seconds; DNA extract was pooled together at the end. DNA was stored at −20°C until further use. The hydrothermal clay is very difficult from which to extract DNA due to not only the complicated clay matrix that can inhibit PCR reactions or bind to DNA, but also the instability of DNA at lower pH or overall degraded nature of DNA from extreme temperatures [10]. For example, the addition of a TE buffer was added to the sediment, as in many cases, and the 0.25 g sediment/clay per extraction tube completely absorbed the initial 60 *μ*L lysis solution.

### 2.2. 16S rRNA Gene Amplification and Sequencing of DNA

Polymerase chain reactions were executed with predispensed, freeze-dried PCR reagents via the Illustra™ puReTaq Read-To-Go PCR Beads (GE Healthcare Life Sciences) to selectively amplify the V6-V9 hypervariable regions of the 16S rRNA gene of archaeal and bacterial species. Selective amplification was performed using the primer pair 906F (5′-AAACTYAAAKGAATTGRCGG-3′) and a modified version of 1392R (5′-ACGGGCGGTGTGTRC-3′) [11], which were modified further with the addition of oligonucleotide adapters used in the 454 sequencing protocol, as well as barcodes to permit numerous samples to be sequenced together and still distinguished in downstream analysis. This modified primer set was initially designed and tested on Dead Sea mesocosm water samples and has successfully shown phylogenetically diverse amplification of both bacterial and archaeal species [11]. We used the following proportions of reagents with the PCR beads: 1.5 *μ*L of forward primer (10 *μ*M), 1.5 *μ*L of reverse primer (10 *μ*M), 10 *μ*L DNA template, and 12 *μ*L sterile water. The mixture was incubated at 94°C for 5 minutes and was followed by 28 cycles of alternating temperatures as follows: 94°C for 1 min, 53°C for 25 s, and 72°C for 2 min. For the following samples, 34 cycles of PCR was used: C0014B-1H-5, C0014B-2H-7, C0014B-4H-8, C0014B-5H-15, and C0014G-5H-3. A final elongation step at 72°C was extended for 20 min. PCR products were gel-purified on a 1% agarose gel using the PrepEase® Gel Extraction Kit (Affymetrix, Inc.) according to the manufacturer's instructions.

Select samples were also sequenced using Illumina technology (Table S1). DNA extract was sent to the Marine Biological Laboratory for all preparation and sequencing. Amplification for the Archaeal V6 region used forward primer 958F (AATTGGANTCAACGCCGG) and reverse primer 1048R (CGRCRGCCATGYACCWC). Individual oligos are mixed in equal proportions for a 10 *μ*M working concentration. Polymerase chain reaction mixture conditions for a 100 *μ*L reaction are as follows: 1x HiFi Buffer, 2 mM MgSO_4_, 0.2 mM dNTPs, 0.3 *μ*M combined primers, 10 units Platinum HiFi, and 5–20 ng template. The mixture was incubated at 94°C for 3 minutes and was followed by 30 cycles of alternating temperatures as follows: 94°C for 30 s, 60°C for 45 s, and 72°C for 1 min. A final extension was held at 72°C for 2 min. All reactions were done in triplicate. The reactions were cleaned and unwanted small products were removed using Qiagen 96-well MinElute plates. The multiplex pools were size-selected by Pippin Prep and quantified using KapaBiosystems qPCR before clustering on the flow cell. Refer to https://vamps.mbl.edu/resources/primers.php for additional information. Data are a part of Projects “DCO_BRA_Bv6” (bacterial samples) and “DCO_BRA_Av6” (archaeal samples).

### 2.3. Analysis of 16S rRNA Gene Amplicons

Sample demultiplexing was performed in Mothur (v.1.30.1), as well as some preliminary quality controls eliminating sequences shorter than 100 base pairs, with more than one mismatch in the barcode sequence, with more than two mismatches in the primer sequence, or with more than eight homopolymers. In addition, sequences were screened by quality score using the “qwindowaverage” function, set at a quality of 35 [12]. These files have been made available on the Metagenomics RAST server (metagenomics.anl.gov) under project name “IODP331_amplicons” with MG-RAST ID accession numbers 4633437.3–4633472.3 (see Table S1) [13].

The resulting individual fasta files were then processed as a single job with the NGS analysis pipeline of the SILVA rRNA gene database project (SILVAngs 1.1) according to the default parameters [14]. This pipeline included alignment with the SILVA Incremental Aligner (SINA v1.2.10 for ARB SVN (revision 21008)) [15] against the SILVA SSU rRNA SEED and quality controlled [14]. Specifically, reads that had fewer than 50 aligned nucleotides and/or more than 2% ambiguities or homopolymers were excluded from further processing, as were putative contaminations, artifacts, and reads with a low alignment quality. The remaining sequences were dereplicated and clustered into operational taxonomic units (OTUs) on a per sample basis, using cd-hit-est (version 3.1.2; http://www.bioinformatics.org/cd-hit/) [16] running in* accurate mode*, ignoring overhangs, and applying identity criteria of 1.00 and 0.98, respectively. Classification was performed by a local nucleotide BLAST [17] search against the nonredundant version of the SILVA SSU Ref dataset (release 115; https://www.arb-silva.de/) using blastn (version 2.2.28+; https://blast.ncbi.nlm.nih.gov/Blast.cgi) with standard settings [18]. Reads were classified if the value of the function “(% sequence identity + % alignment coverage)/2” exceeded 93. Classification of each OTU reference read was mapped onto all reads that were assigned to the respective OTU. Those that did not fall within this classification quality were assigned to the group “No Relative.” Classifications of the reference sequences were then mapped onto reads within their respective OTUs.

The Illumina dataset was analyzed as a part of the Visualization and Analysis of Microbial Populations Structure (VAMPS) [19]. An additional assessment of the dataset used the SilvaNGS 1.1 pipeline as described in the previous paragraph [14]. See Supplemental Discussion for further results.

The maximum likelihood tree in Figure 4 was made by aligning the full-length reference sequences in MEGA and exporting the alignment to PAUP. The sequences were run through a branch-and-bound search with random seed numbers, and this base topology was used as a constraint on which the shorter ANME-1 sequences from the C0014B-2-10 analysis are added. The three sequences displayed were condensed down from seven C0014B-2-10 sequences, which were nearly identical. They were combined such that ambiguous base pairs were used to adjust the few differences in base pairs.

### 2.4. Analysis of Geochemical Data

Porewater chemistry data (i.e., sulfate, methane, alkalinity, and potassium concentrations in Figure S2) were downloaded from the SIO7 Data Center (http://sio7.jamstec.go.jp/) that aims to distribute the science data acquired by International Ocean Discovery Program and Integrated Ocean Drilling Program expeditions of D/V Chikyu. The scientific data recorded are from the J-CORES database. The temperature data points were referenced in the IODP Expedition 331 Proceedings [7]. The samples analyzed for *δ* ^13^CH_4_ came from sediment plugs stored in gas-tight storage vials. Gas samples were analyzed using a HP 5890 Series II GC with a flame ionization detector and a custom vacuum inlet system. Daily standard curves are generated using appropriate standards from Scott Specialty Gases. Analytical precision for these samples is better than ±2%. ~5 nmoles of analyte was injected into a helium carrier stream and purified using a modified PreCon peripheral device before analysis on a Delta V mass spectrometer [20]. External precision on this technique is ±0.3‰ with daily standards providing the means of accurately reporting data directly on the VPDB scale. Modifications to the system correct for the recently discovered Kr interference during *δ* ^13^CH_4_ analyses with an additional chromatography step after the combustion of CH_4_ to CO_2_ to separate Kr from CO_2_ [21].

### 2.5. Correlation Analysis

The software package IBM® SPSS® Statistics Version 24 was used to run bivariate correlations between dominant microbial taxa and geochemical parameters, using the defaults settings for* Correlation* function. Analyses used relative proportions of taxa available from the 0.30 to 44.85 mbsf. Samples for microbiological and geochemical analyses on IODP Expedition 331 did not come from the same core section. Due to this offset, we used the geochemical information that corresponded closest to the depth of the microbiological sample. The tests produced Pearson Correlation Coefficients and associated significance values (from a 2-tailed test) for each pair of variables tested. The data reported here are only those corresponding to a significance of <0.01.

## 3. Results and Discussion

### 3.1. Domains of Life Represented in the Subsurface

**Figure 1:**
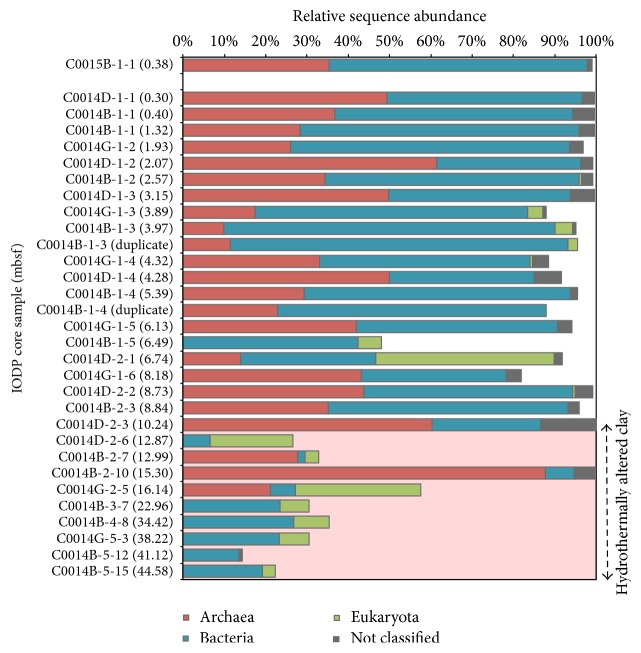
Relative sequence abundance, listed as % across the *x*-axis, of 16S rRNA gene amplicons from Site C0014 sediment samples classified at the domain level: Archaea, Bacteria, Eukaryota, and not classified. IODP sample names are listed along the *y*-axis by increasing depth (meters) below seafloor, depth in parentheses. Site C0015, 600 m northwest and upslope of the hydrothermal vent (shown separately as the topmost sample), showed no current hydrothermal activity and is being compared to represent nonhydrothermal conditions within the Iheya North Field. The remainders of the horizontal bars represent the relative abundance of sequences in that sample consistent with those found in the drilling fluid [1] and/or extraction blanks and are excluded here, as they are less likely to represent indigenous taxa. See Supplemental Table S1 for sequence information. The red shaded region corresponds to horizons composed of a hydrothermally altered clay lithology.

From recovered genomic DNA, 28 distinct sediment samples (two samples in duplicate) ranging from the surface to 44.58 mbsf from IODP Expedition 331 Site C0014 were selectively amplified and sequenced for 16S rRNA gene analysis using 454 technology and subsequently classified for taxonomic identification. The amplicon data in Figure 1 is presented as a percent relative sequence abundance of classified* Archaea*,* Bacteria*,* Eukaryotes*, and* Not classified* sequences for each sampled depth horizon, as a proportion from the total sequence yield (see Table S1 for total sequence yield). The bars in Figure 1 generally represent many known subsurface groups, many of which are Archaea, in the upper 16 meters of the sediment column. The dataset also shows isolated peaks in the relative abundance of archaeal sequences to bacterial sequences at depths 10.24, 12.99, and 15.30 mbsf (Figure 1). For comparison, IODP Expedition 331 Site C0015, 600 m northwest and upslope of the hydrothermal vent, showed no current hydrothermal activity and was, in this study, shown separately as a control to represent nonhydrothermal conditions within the Iheya North Field. The remainders of the bar graph in Figure 1 represents sequences consistent with those found in the drilling fluid [1] and/or extraction blanks (see Identification of External or Background DNA in the Supplementary Material for details) and are excluded from further analyses, as they might not be indigenous.

In this study, consistent with these challenging samples, there is an increased proportion of sequences that matched sequences from contaminants in drilling fluid and laboratory extraction blanks in the deeper horizons. This trend is observed beginning at the 16.14 mbsf horizon, where the remainders of the bar graph represent a higher percent of recoverable DNA sequences than in shallower horizons (Figure 1). Additionally, bivariate correlations between the relative proportion of taxa from each sediment horizon and corresponding environmental variables demonstrate the strongest positive, 0.864 and 0.860, statistically significant (*p* < 0.01, 2-tailed test) correlation between these “nonindigenous sequences” with temperature and depth, respectively (Table 1). Eukaryotic sequences also appear in most horizons, despite the archaeal and bacterial specificity of the 16S rRNA primers, with several horizons in significantly high relative abundance represented primarily by Mollusca (6.74 mbsf), Basidiomycota (12.87 mbsf), and Ascomycota (16.14 mbsf) (Figure S5). Although we did not anticipate that eukaryotic DNA would amplify with our 16S rRNA specific primer set, these sequences most likely represent a combination of amplified indigenous and/or relict environmental DNA. For example, Edgcomb et al. [22] and Orsi et al. [23] have demonstrated that the marine subsurface down to at least 35 mbsf in marine sediments is occupied by living eukaryotes, primarily fungi (*Basidiomycetes*), as well as ancient (2.7 Myr) eukaryotic genomic material (diatoms, Viridiplantae, Alveolata, and Fungi). Also below 16.14 mbsf, there are recovered sequences that likely indicate a signal for terrestrial runoff and environmental DNA. Interestingly, archaeal sequences were not detected in contamination assessments [1]. Thus, we interpret archaeal DNA to be representative of an indigenous microbial community in this study. We are, therefore, skeptical of the remaining bacterial sequences and extant terrestrial DNA found in the datasets from five deepest horizons, and, at present, we conclude that these horizons do not have substantial microbial communities. Overall, credible bacterial 16S rRNA gene amplicons, and any archaeal 16S rRNA gene amplicons, could not be recovered below 16.14 mbsf. Thus, we cannot confidently make detailed conclusions regarding specific taxonomic shifts in the subsurface biosphere below 16.14 mbsf (*in situ* temperature of ca. 55°C) due to the much-reduced DNA yield near this discontinuity.

### 3.2. Subsurface Prokaryotic Diversity

**Figure 2:**
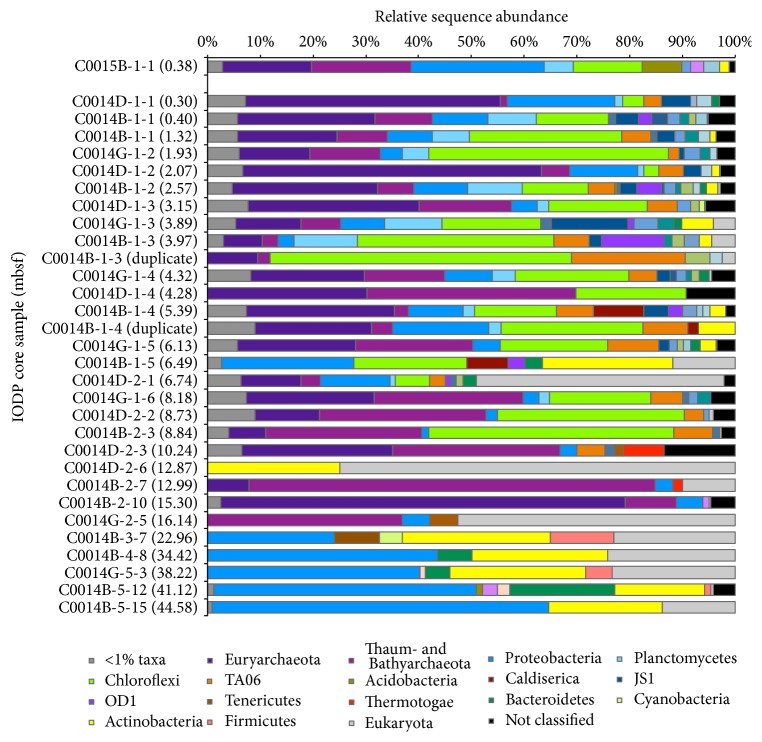
Relative sequence abundance, listed as % across the *x*-axis, of 16S rRNA gene amplicons from Site C0014 sediment samples classified at the phylum level. Sample horizons are listed by increasing depth below seafloor. IODP sample names are listed along the *y*-axis by increasing depth (meters) below seafloor, depth in parentheses. Site C0015, 600 m northwest and upslope of the hydrothermal vent (shown separately as the topmost sample), showed no current hydrothermal activity and is being compared to represent nonhydrothermal conditions within the Iheya North Field. Sequences included here are those identified as likely to represent indigenous taxa in Figure 1. See Table S1 for additional sequence information.

The relative abundances of bacterial and archaeal amplicons interpreted at the phylum level (Figure 2) reveal a diverse community with distinct community shifts toward higher* in situ* temperatures. For example, the various horizons studied in the top 8.84 mbsf at IODP Expedition Site C0014 show similarities in their most highly represented phyla (e.g., Euryarchaeota, Thaum- and Crenarchaeota, Proteobacteria, Planctomycetes, Chloroflexi, and TA06), and they all show a broad similarity to the C0015 control horizon. However, the Chloroflexi sequences, which appear frequently in oceanic subsurface sediments [24], are consistently represented as a significant phylotype throughout this interval but are nearly absent at 10.24 mbsf and are not present in deeper horizons. The phyla Bacterial Candidatus TA06 and Planctomycetes follow a similar trend as Chloroflexi with less overall abundance and also abruptly disappear beyond 10.24 mbsf. Bivariate correlations between the relative proportions of taxa from each sediment horizon shown in Table 1 demonstrate this significant (*p* < 0.01, 2-tailed test) correlation between Chloroflexi and TA06 sequence abundances. Similarly, a high diversity of less abundant bacterial phyla is observed at Site C0014, but only in samples from above 10.24 mbsf. Samples C0014B-1-5 and C0014D-2-6 are unique from their surrounding horizons in that they yielded no archaeal sequences. While the 6.49 and 12.87 mbsf do not show any geochemical or lithological anomalies to infer potentially unique microbial niches, we can only speculate that we have not captured the entire sample diversity in these horizons due to deficient microbial biomass, or that no archaeal community exists at these horizons. The abundance of microbial cells reported from IODP Expedition 331 Site C0014 indicate detectable (on the order of 10^6^–10^8^ cells/mL sediment) cells down to 2.35 mbsf in Hole B (except C0014B-2-10, approximately 15.3 mbsf, where cells were detected) and 10.17 mbsf in Hole D [7]. Thus, reduced microbial assemblages at depth as well as method limitations make DNA recovery more tenuous. Deeper in the section, we observe higher relative abundances of DNA amplicons from euryarchaeotic taxa at 12.99 and 15.30 mbsf and “Cren- and Thaumarchaeota” at 15.30 mbsf. Additionally, the phylum Thermotogae becomes more abundant at 10.24 and 12.99 mbsf, which may represent a shift toward more optimal conditions for this largely thermophilic phylum. Because the modified primer set appeared to successfully amplify both Euryarchaeota and Cren- and Thaumarchaeota sequences consistently through most horizons above 16.14 mbsf, we do not interpret the signals from 10.24, 12.99, and 15.30 mbsf to be a consequence of primer and/or amplification bias. Furthermore, detectable microbial cells from ~15 mbsf indicate that a substantial microbial assemblage contributes to the DNA recovered.

The discontinuation of Chloroflexi and TA06 phyla and general loss of broad microbial diversity below the 10.24 mbsf sample, interestingly, corresponds with a change in clay lithology. Both Chloroflexi and TA06 phyla show significant correlations with depth and temperature but more so with K^+^, NH_4_^+^, and Mg^+2^, which are associated with uptake or exchange of chemical species by changes in clay mineralogy (Figure S2(F)). The nonhydrothermal hemipelagic ooze shifts to a hydrothermally altered mottled pale gray with alteration products illite and montmorillonite over the course of 9–12 mbsf [7]. Although the taxonomic richness seems to be affected by the geological and geochemical boundary, archaeal DNA sequences appear to dominate through the transition from temperate to hydrothermal conditions. Overall, our results strongly suggest that this lithologic and temperature transition represents a considerable obstacle for the survival of Chloroflexi and other rare taxa, while certain archaeal taxa are able to persist several meters deeper in hydrothermal clay with an* in situ *temperature of approximately 33°C.

### 3.3. Shifts in Subsurface Archaeal Relative Abundance

**Figure 3:**
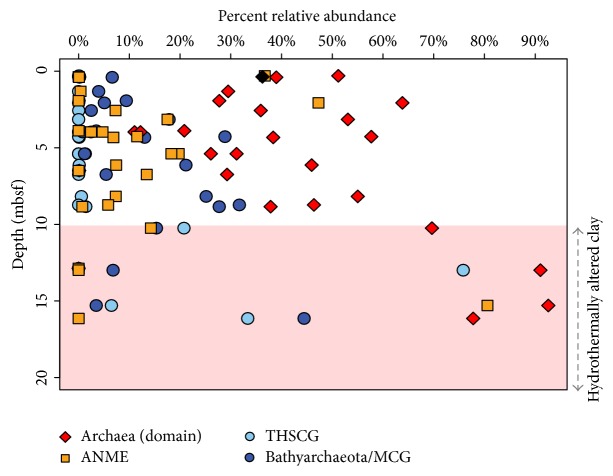
Relative abundance of archaeal (domain) sequences (red diamonds) and three featured subclassifications of archaeal taxa. The percentage values were calculated as a proportion of “indigenous” prokaryotic sequences, which excludes eukaryotic, not classified, and nonindigenous bacterial sequences. The black diamond represents the sample from Site C0015. Anaerobic methanotrophic archaea (ANME, orange squares) are classified within the Euryarchaeota phylum, while the MCG/Bathyarchaeota (dark blue circles) and Terrestrial Hot Spring Crenarchaeotic Group (THSCG, light blue circles) are within the Thaumarchaeota. Archaeal sequences were not detected below 16.14 mbsf in this study. The red shaded region corresponds to horizons composed of a hydrothermally altered clay lithology.

Of the total indigenous Prokaryotic sequences within the top 16.14 mbsf at IODP Expedition 331 Site C0014, the recovered archaeal sequences (domain level) increase in relative abundance with depth (Figure 3, red diamonds). The bivariate correlation analysis calculated a significant, but weak correlation between the relative proportion of total archaeal sequences recovered and depth and temperature. However, the archaeal relative abundance in the correlation analysis was calculated as a proportion of total sequences recovered. Figure 3, on the other hand, is intended to decouple the indigenous prokaryotic sequences from background noise and illustrates a clearer relationship between the proportions of archaeal sequences as a function of depth. Notably, below 10.24 mbsf there is a marked increase in relative abundance in archaeal sequences, reaching up to 92%, in all but one of the deepest horizons (Figure 3). The Site C0015 sample (0.37 mbsf, black diamond) showed an indigenous archaeal fractional abundance of 36% (Figure 3), which is similar to the surface sediments of Site C0014. Even though the deepest Site C0014 horizon in which Archaea are found (16.14 mbsf) yields considerably fewer total indigenous sequences, the relative abundance of archaeal sequences is still significantly higher than its surface counterparts. Until recently, Archaea in the marine subsurface were considered to represent an insignificant portion of the active subsurface community [25]. However, the data in this study, like more recent findings from Biddle et al. [26], and Teske and Sørenson [27], suggest that the subsurface contains a community with a potentially significant contribution of Archaea. The highest relative abundance of Archaea at 92% occurs at 15.30 mbsf (Figure 3), corresponding to an estimated temperature of 55°C (Table S1). Archaeal sequences represent 78% abundance in the subsequent sample at 16.14 mbsf (Figure 3) but are not present at all in the sequencing results from deeper horizons. Ten additional efforts to recover and amplify sequences from samples below 16.14 mbsf failed (see Table S1 for details). These deeper samples between 10.24 and 16.14 mbsf have* in situ* temperatures approaching 57°C (Table S1) and suggest a shift in community toward archaeal thermophiles.

### 3.4. Shifts in Subsurface Archaeal Taxa

Figure 3 shows the Bathyarchaeota (formerly Miscellaneous Crenarchaeotic Group) (dark blue circles) and Terrestrial Hot Spring Crenarchaeotic Group (THSCG) from the Thaumarchaeota phylum (light blue circles). Both taxonomic groups exhibit an increase in relative abundance within the deeper horizons at IODP Expedition 331 Site C0014. Members of the highly diverse Bathyarchaeota are globally distributed in various marine and continental environments. Recent information on the ecological role of Bathyarchaeota archaea has revealed a diverse subgroup of organoheterotrophic and autotrophic acetogens that are capable of degrading complex carbohydrate polymers of photosynthetic origin, low-molecular weight carbon substrates, and proteins [28–32] and have the cellular machinery for a methane metabolism [33]. The widespread abundance of Bathyarchaeota throughout Site C0014 (Figures 3 and S3) suggests that they could be less affected by the increasing temperature than other bacterial taxa or could represent persistent, relict DNA. Similarly, the THSCG become more abundant, particularly below the 8.84 mbsf horizon (Figure 3), where Archaea represent the majority of indigenous sequences. In the sample from 12.87 mbsf, THSCG represent ~80% of archaeal sequences. No cultured representatives have been studied from the THSCG; however, the documented samples comprising this clade come from a 1–10 cmbsf, 100°C sediment layer within a middle Okinawa Trough hydrothermal field [34]. Additionally, sequences from THSCG have also been documented in the sediments from Iheya North Hydrothermal Field Site C0017, or the presumed site of recharging seawater [1]. Yanagawa et al. showed evidence of THSCG at 141 mbsf, corresponding to 83°C, which is optimal for a hyperthermophilic community. Therefore, it is somewhat surprising that we detected THSCG sequences at a 55°C sediment horizon (Site C0014, 12.87 mbsf). It is possible that our detection of THSCG could be a microbial relict from a time when this horizon experienced a hotter temperature fluctuation. The appearance of THSCG restricted to these deeper horizons, however, demonstrates the recent establishment of a niche community in response to environmental conditions and supports our first hypothesis in which mesophilic, marine subsurface taxa have been replaced by a high temperature adapted microbial population.

**Figure 4:**
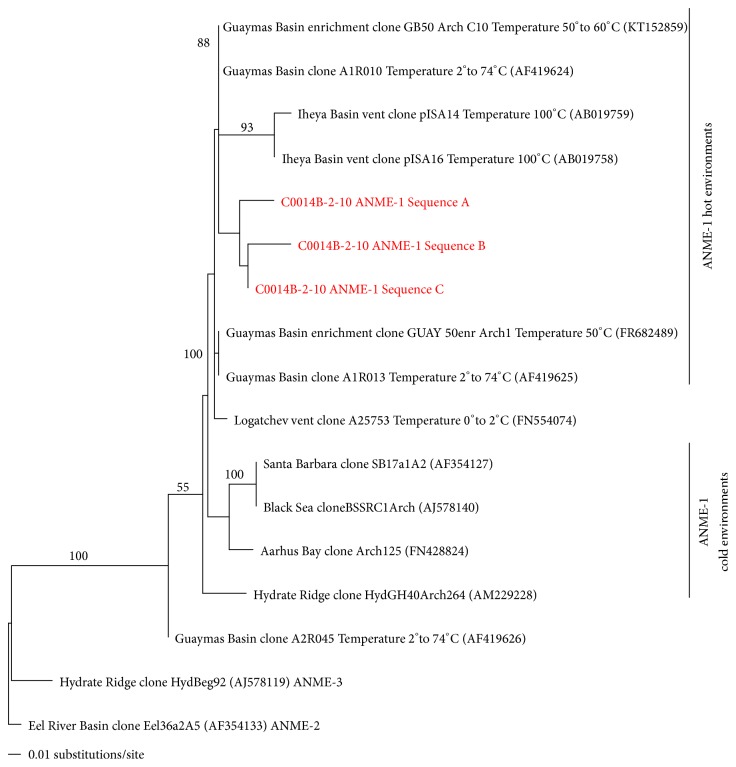
Maximum likelihood constraint tree in which full length 16S rRNA gene reference sequences (black) represent a base topology on which the amplicon sequences corresponding with ANME-1 from C0014B-2-10 are mapped (red). Bootstrap support is shown on full-length sequences from the initial full-length analysis. In parentheses are the NCBI accession identifiers.

Also shown in Figure 3 are the relative abundances observed of DNA amplicons representing the anaerobic methanotrophic archaea (ANME, orange squares). ANME are members of a microbial consortium involved in the anaerobic oxidation of methane (AOM) in anoxic marine sediments [35]. Generally, driven by sulfate reduction, the anaerobic oxidation of methane is a critical control on the flux of methane from marine sediments to the atmosphere. Members of these microbial consortia have not yet been isolated, but archaeal subgroups ANME-1 and ANME-2 have been found to be related to methanogenic Archaea of the Methanosarcinales and Methanomicrobiales and are often found in association with sulfate-reducing bacterial counterparts [35, 36]. The anaerobic oxidation of methane is a significant process in coastal marine sediments, and this process is recently getting more attention as part of the trophic ecology of vent ecosystems (e.g., Guaymas Basin, Gulf of California, Mexico) [37–39]. The sequences at the 15.30 mbsf horizon represent 81% of the total indigenous sequences. Though the relative abundances of ANME are highly variable throughout the sediment profile at Site C0014 (Figures 3 and S3), the highest relative abundance of ANME in the 15.30 mbsf (c.a. 55°C) suggests a potential methane-oxidizing niche in the thermophilic regime. Further taxonomic evidence of a thermophilic methane-oxidizing taxon is seen in Figure 4, where sequences from the C0014B-2-10 horizon were aligned with other published ANME-1 sequences. In order to best resolve the taxonomy of the C0014B-2-10 ANME sequences, full-length 16S rRNA gene alignments of reference sequences were generated as a base topology constraint on which the shorter amplicons are mapped. The ANME-1 sequences generally cluster based on temperature regime (i.e., hot and cold environments). ANME-1 sequences from C0014B-2-10 group are the most close to other Iheya Basin clones from high temperature enrichments and are also part of a larger clade with several clones from Guaymas Basin hydrothermal sediments. Thus, the abundant ANME-1 representatives from 15.30 mbsf at Site C0014 appear more similar to high temperature adapted methanotrophs than those found in cold, methane-seep type environments and indicate a likely abundant and possibly active thermophilic methane-oxidizing community.

Overall, geochemical evidence also supports the presence of biological AOM in the top ~15 m of the sediment profile at Site C0014. In the topmost 5 mbsf, which correspond to an estimated temperature range between 5 and 20°C, there is an inverse relationship between methane and sulfate concentrations that is accompanied by an increase in alkalinity (Figure S2). Generally, sulfate-methane transition zones provide a niche for microbially driven sulfate-dependent methane oxidation, a process which produces alkalinity [40]. ANME sequences are also found in high relative abundances in several of these shallower horizons. Below the peak in methane concentration at ~4 mbsf is an overall decrease over the subsequent 4 m, indicating methane consumption. One gas headspace measurement at 1.4 mbsf and two at 7.8 mbsf show an enrichment in *δ* ^13^CH_4_ of 10.1–11.7‰ relative to the that of the presumed source gas horizons (Figure S2(B, C) and Table S2). These suspected thermogenic horizons were based on three void gas (extreme degassing of core) measurements at 19.22, 21.36, and 24.81 mbsf and were considered to represent the source gas (an average of −56.27*‰*) throughout the sediment profile in this study (Figure S2(C) and Table S2). These geochemical observations in the Site C0014 sediment profile are consistent with biological AOM, where biological methane consumption leaves an enrichment of ^13^C in the remaining methane relative to its source value [41]. Elevated concentrations of sulfate coincide with the two horizons showing enriched *δ* ^13^CH_4_ values ranging between −44.5 and −46.1‰ (Figure S2). Pumice lenses have been documented throughout C0014 cores and can provide conduits for seawater transport. However, we interpret this source of entrained seawater as a necessary source of sulfate for biological anaerobic methane oxidation in surrounding horizons of otherwise uniformly, low-conductivity marine clay. Observed ANME sequences throughout this unit, and the particularly high relative abundances at 2.07 mbsf, 5.39 mbsf, and 6.74 mbsf (Figures 3 and S3), complement the geochemical evidence in support of an active AOM biosphere. The subsequent 10–15 mbsf horizons, corresponding to a temperature range of 15–55°C, are also accompanied by a decrease in methane concentration with depth, which could be associated with methane consumption. The ANME sequences associated with the 15.30 mbsf horizon are from thermophilic taxa (Figure 4) and represent 81% of total sequences (Figure 3). Though the taxonomic evidence indicates a thermophilic methane-oxidizing niche, the methane isotope data suggests this community may not be currently wholly active. Beyond 16.14 mbsf, a potential AOM zone at ~27 mbsf is hypothesized from a decrease in methane concentration and an enrichment of *δ* ^13^CH_4_ relative to that of source methane (Figure S2(B, C)). However, based on the lack of DNA recovery at this depth and an estimated temperature of 96°C, we cannot conclude that this horizon hosts an active biosphere. There are also no reported pumice clasts in this sample horizon to suggest an intrusion of seawater, where *δ* ^13^CH_4_ values of seawater have been reported between −52.0 and −48.2‰ [42]. Rather, abiotic AOM could be occurring at or near this depth.

## 4. Conclusions

The Okinawa back-arc basin is a unique environmental setting to analyze microbial communities through a range of temperatures because of its subsurface hydrothermal network within continental margin-like sediments. This study represents a proxy for the distribution and extent of life in other subsurface environments, where hotter temperatures are more difficult to reach at greater depths. Based on the taxonomic information in this study, the microbial community in the deeper, hotter hydrothermal clay horizons of IODP Expedition Site C0014 is distinct from the shallower, cooler horizons. In this study, we use archaeal sequences as a confident and conservative estimate of the extent of the biosphere, which extends in this profile down to 16 mbsf. Sequences below, particularly those indicative of relict plant material, suggest that the diminished sequence yield from the indigenous population is dampened by such signals and make detailed conclusions about an indigenous community more tenuous.

The overall heterogeneous community composition in these sediments exhibits similarities to other studied marine sediments, and the results of this study complement the findings from Yanagawa and colleagues [43]. Many taxa identified here, for example, Chloroflexi and Bathyarchaeota, have ubiquitous distributions in marine subsurface habitats. The widespread distribution of the cosmopolitan Bathyarchaeota persisting into hydrothermal clay likely suggests an ecophysiological flexibility within a wide range of temperature and geological conditions but could also represent relict DNA. Chloroflexi appear to be restricted to the upper horizons associated with nonhydrothermal marine mud, which reflects a general geochemical, lithological, and/or temperature boundary to much of the microbial diversity. The isolated peaks in abundances of archaeal sequences at depth indicate a recent establishment of potentially better-adapted archaeal community to the prominent hydrothermal conditions over other taxa. The uncultured archaeal taxa identified here (THSCG and thermophilic ANME-1) that are observed here in significant proportion in the hydrothermal clay horizons are also observed in other high temperature sediments (Okinawa Trough and Guaymas Basin) and suggest that high temperature microbial communities may be biogeographically similar among other sedimented hydrothermal vent ecosystems. The appearance of these high temperature taxa supports our first hypothesis and demonstrates a transition from mesophilic marine taxa in the top 10 m to the recent establishment of a temperature adapted community restricted to the deeper, hotter horizons. This taxonomic dataset, in combination with geochemical and isotopic data, also suggest that methanotrophy may have once been a significant process occurring in these subsurface sediments, particularly in the thermophilic regime. The subsurface hydrothermal system in the Iheya North Hydrothermal Field is very dynamic and reflects a diverse subsurface biosphere seemingly adapted to the range of conditions experienced through a hydrothermal gradient, both low and high temperature. Overall, these results support a conceptual model in which a community of cosmopolitan marine subsurface bacteria (e.g., Chloroflexi) persists until a lithological/geochemical boundary, but other cosmopolitan archaeal taxa (i.e., Bathyarchaeota) persist further into the hydrothermal clay. Additionally, the hydrothermal clay horizons show isolated peaks in abundances of specific high temperature archaeal phylotypes (e.g., thermophilic ANME and THSCG) that suggest the recent establishment of high temperature adapted microbial niches maybe have once been supported under different environmental conditions. Lastly, there is also relict DNA in horizons where the conditions suitable for certain communities have ceased (ANME, THSCG, Bathyarchaeota, and plant DNA).

## Supplementary Material

The Supplementary Material includes detailed sampling and sequencing information from this study, environmental data associated with Site C0014, and discussion of an additional dataset from a separate sequencing attempt. Geochemical plots and methane isotope data are also included.

## Figures and Tables

**Table 1:** Bivariate correlation values of environmental variables with significant correlation to dominant taxa (second column); taxa that have significant correlations with one another (third column); and environmental variables that have significant correlation with depth (bottom row). The chemical species abbreviation or taxon name is listed with the correlation value in parentheses. All correlation values listed here are significant at the 0.01 level (2-tailed).

	Environmental variables	Other phyla
Archaea (domain)	Br^−^ (−0.467), temperature (−0.502), depth (−0.500)	

Nonindigenous sequences	B (0.755), Ba^+2^ (0.525), Ca^+2^ (0.721), K^+^ (0.725), Li^+^ (0.701), Mg^+2^ (−0.796), Mn (0.788), NH_4_^+^ (0.765), Na^+^ (−0.586), Rb^+^ (0.692), SO_4_^−2^ (−0.533), Si (0.690), temperature (0.864), depth (0.860), pH (−0.540)	

Deep Sea Hydrothermal Vent Group 6 (DHVEG-6)	Ca^+2^ (−0.535), K^+^ (−0.504), Li^+^ (−0.512), Mg^+2^ (0.504), NH_4_^+^ (−0.554), pH (−0.601), temperature (−0.541), depth (−0.551)	Planctomycetes (0.551)

ANME-1		

Miscellaneous Crenarchaeotic Group (Bathyarchaeota)	Ca^+2^ (−0.484), SO_4_^−2^ (0.619)	Proteobacteria (−0.497)

Terrestrial Hot Spring Crenarchaeotic Group (THSCG)		

Bacteroidetes	Ba^+2^ (0.533), Mn (0.647), temperature (0.561)	Proteobacteria (0.580)

Chloroflexi	Ca^+2^ (−0.502), K^+^ (−0.562), Li^+^ (−0.583), Mg^+2^ (0.608), Mn (0.528), NH_4_^+^ (−0.607), SO_4_^−2^ (0.463), temperature (−0.537), depth (−0.513)	TA06 (0.677)

Proteobacteria	Ca^+2^ (0.636), Ba^+2^ (0.615), K^+^ (0.593), Li^+^ (0.523), Mg^+2^ (−0.683), Mn (0.735), NH_4_^+^ (0.613), Na^+^ (−0.547), SO_4_^−2^ (−0.520), temperature (0.813), depth (0.825)	

Planctomycetes		

TA06	K^+^ (−0.501), Li^+^ (−0.512), Mg^+2^ (0.544), NH_4_^+^ (−0.536), temperature (−0.469)	

Depth	Ca^+2^ (0.757), Ba^+2^ (0.558), K^+^ (0.774), Li^+^ (0.745), Mg^+2^ (−0.889), Mn (0.885), NH_4_^+^ (0.836), Na^+^ (−0.692), SO_4_^−2^ (−0.581), pH (−0.500), temperature (0.997)	
