# Supplementary material for: Marine Subsurface Microbial Community Shifts Across a Hydrothermal Gradient in Okinawa Trough Sediments
Source: Archaea. 2016 Dec 20;2016:2690329. doi: 10.1155/2016/2690329 (PMC5206410; doi:10.1155/2016/2690329)
Supplement: Supplementary file 1 — The Supplementary Material includes detailed sampling and sequencing information from this study, environmental data associated with Site C0014, and discussion of an additional dataset from a separate sequencing attempt. Geochemical plots and methane isotope data are also included. [file 2690329.f1.docx]

**Supporting Information**

**
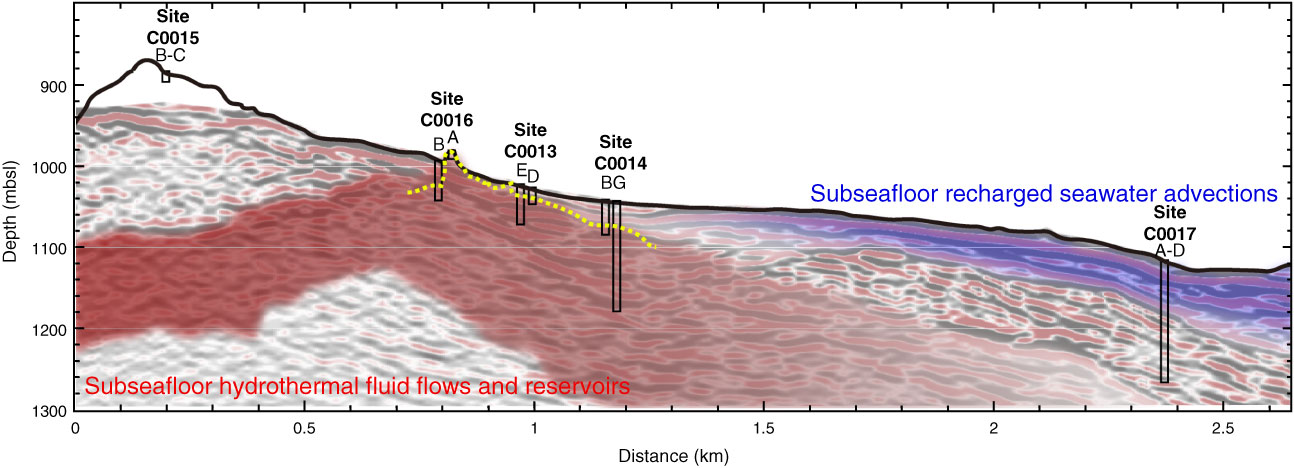
**

**Figure S1** (source: Takai *et al.*, 2011) Schematic illustration of the possible spatial extent of subseafloor hydrothermal fluid flow and reservoirs (red) and recharged seawater advection (blue) based on preexisting seismic interpretation and drilling results from Expedition 331. Bars indicate the deeper holes drilled during Expedition 331 and their total penetration depths. The yellow dotted line denotes the shallowest depth of high temperatures of hydrothermal fluids we encountered during the expedition. Hole depths are fit to the seismic reflection profile assuming that 1 s two-way traveltime = 750 m.

**Table S1** Sample information, which includes IODP sample number, corresponding depth, sequencing yields, and sedimentary and temperature information. Samples with less than 16 sequences (the extraction blank yield) were not used in further analyses. Samples with no amplification were not sequenced. Temperature measurements through 55°C taken on IODP Expedition 331 used an ATCP3 temperature shoe attached to the hydraulic piston coring system core barrel, while temperature measurements in deeper core section used thermoseal temperature-sensitive strips (K. Takai et al., 2011). Sample depths referred to in the discussion of the paper used the average of Top and Bottom depths.

| **IODP Sample Number** | **Top Depth DSF, MSF, WSF and CSF-A (mbsf)** | **Bottom Depth DSF, MSF, WSF and CSF-A (mbsf)** | **Samples with no amplification from PCR** | **454 Sequencing Yield (V6-V9 universal primers)** | **Illumina Sequencing Yield: Bacterial; Archaeal (V6 bacterial and archaeal primers)** | **Sediment Type** | **Estimated Temperature based on 3.3** °**C/m gradient** | **Measured Temperature** | **MG-RAST accession number** |
| --- | --- | --- | --- | --- | --- | --- | --- | --- | --- |
| C0015B-1H-1, 30.0-45.0 cm | 0.300 | 0.450 |  | 4850 |  | Pumiceous Gravel |  |  | 4633466.3 |
| C0014D-1H-1, 23.0-38.0 cm | 0.230 | 0.380 |  | 3349 | 646766;  554173 | Clay | 5.2 |  | 4633456.3 |
| C0014G-1H-1, 28.0-40.0^#^ | 0.28 | 0.40 |  |  |  | Clay | 5.4 |  |  |
| C0014B-1H-1, 35.0-45.0 cm | 0.350 | 0.450 |  | 5437 | 592568;  757769 | Silty Clay | 5.6 |  | 4633437.3 |
| C0014B-1H-1, 122.0-142.0 cm | 1.220 | 1.420 |  | 1848 |  | Clay | 8.5 |  | 4633452.3 |
| C0014G-1H-2, 45.0-57.0 cm | 1.870 | 1.990 |  | 6287 |  | Silty Clay | 10.7 |  | 4633471.3 |
| C0014D-1H-2, 57.0-77.0 cm | 1.970 | 2.170 |  | 1739 |  | Sandy Silt | 11.0 |  | 4633464.3 |
| C0014B-1H-2, 110.0-120.0 cm | 2.520 | 2.620 |  | 4637 | 289603;  341982 | Clay | 12.8 |  | 4633438.3 |
| C0014D-1H-3, 27.0-42.0 cm | 3.075 | 3.225 |  | 1024 | 351767;  633536 | Pumiceous Gravel - Matrix Supported | 14.6 |  | 4633442.3 |
| C0014G-1H-3, 100.0-112.0 cm | 3.830 | 3.950 |  | 514 |  | Sandy Clay | 17.1 |  | 4633446.3 |
| C0014B-1H-3 (454 duplicate), 105.0-127.0 cm | 3.860 | 4.080 |  | 141/44 | 292952;  95121 | Clay | 17.2 |  | 4633439.3; 4633457.3 |
| C0014G-1H-4, 0.0-20.0 cm | 4.225 | 4.425 |  | 3360 |  | Pumiceous Gravel - Matrix Supported | 18.4 |  | 4633447.3 |
| C0014D-1H-4, 0.0-10.0 cm | 4.225 | 4.325 |  | 60 | 179358;  596865 | Pumiceous Grit | 18.4 | 21 | 4633469.3 |
| C0014B-1H-4 (454 duplicate), 110.0-122.0 cm | 5.330 | 5.450 |  | 242/292 | 341807;  35808 | Clay | 22.1 |  | 4633440.3; 4633458.3 |
| C0014G-1H-5, 42.0-57.0 cm | 6.060 | 6.210 |  | 2765 |  | Hydrothermal Clay - Horizon with hydrothermal origin | 24.5 |  | 4633472.3 |
| C0014B-1H-5, 75.0-95.0 cm^*^ | 6.390 | 6.590 |  | 1901 |  | Silty Clay | 25.6 | 22 | 4633459.3 |
| C0014D-2H-1, 17.0-32.0 cm | 6.670 | 6.820 |  | 1389 | 241181;  42238 | Pumiceous Gravel - Clast Supported | 26.5 |  | 4633470.3 |
| C0014G-1H-6, 105.0-120.0 cm | 8.100 | 8.250 |  | 5614 |  | Pumiceous Gravel - Clast Supported | 31.2 |  | 4633448.3 |
| C0014D-2H-2, 75.0-95.0 cm | 8.630 | 8.830 |  | 837 | 176555;  297037 | Pumiceous Gravel - Matrix Supported | 33.0 |  | 4633467.3 |
| C0014B-2H-3, 20.0-35.0 cm | 8.770 | 8.920 |  | 2045 | 270289;  203916 | Pumiceous Grit - Matrix Supported | 33.4 |  | 4633460.3 |
| C0014D-2H-3, 88.0-103.0 cm | 10.170 | 10.320 |  | 1809 | 237182;  370577 | Gradation from Clayey Hydrothermal Sand with Mineralized Material to Pumiceous Grit | 38.1 | 15 | 4633468.3 |
| C0014D-2H-4, 66.0-81.0 cm | 11.380 | 11.530 |  | 13 |  | Hydrothermal Clay - Horizon with hydrothermal origin | 42.0 |  | 4633443.3 |
| C0014D-2H-6, 19.0-34.0 cm | 12.795 | 12.945 |  | 70 |  | Hydrothermal Clay - Horizon with hydrothermal origin | 46.7 |  | 4633444.3 |
| C0014B-2H-7, 50.0-70.0 cm^*^ | 12.890 | 13.090 |  | 2403 |  | Hydrothermal Clay - Horizon with hydrothermal origin | 47.0 |  | 4633461.3 |
| C0014B-2H-10, 30.0-44.0 cm | 15.225 | 15.365 |  | 670 | 168476;  364439 | Hydrothermal Clay - Horizon with hydrothermal origin | 54.7 |  | 4633462.3 |
| C0014G-2H-5, 127.0-142.0 cm | 16.065 | 16.215 |  | 34 |  | Hydrothermal Clay - Horizon with hydrothermal origin | 57.5 | 55 | 4633449.3 |
| C0014B-3H-2, 62.0-77.0 cm | 17.460 | 17.610 |  | 0 |  | Hydrothermal Clay - Horizon with hydrothermal origin | 62.1 |  |  |
| C0014E-1H-4, 70.0-90.0 cm | 19.670 | 19.870 |  | 8 |  | Hydrothermal Clay - Horizon with hydrothermal origin | 69.4 |  | 4633445.3 |
| C0014B-3H-5, 0.0-20.0 cm | 19.855 | 20.055 |  | 12 |  | Hydrothermal Clay - Horizon with hydrothermal origin | 70.0 |  | 4633441.3 |
| C0014G-3H-2, 65.0-81.0 cm | 19.990 | 20.150 | x |  |  | Hydrothermal Clay - Horizon with hydrothermal origin | 70.5 |  |  |
| C0014B-3H-7, 99.0-119.0 cm^*^ | 22.865 | 23.065 |  | 2194 |  | Hydrothermal Clay - Horizon with hydrothermal origin | 80.0 |  | 4633463.3 |
| C0014B-3H-9, 47.5-67.5 cm | 24.760 | 24.960 | x |  |  | Hydrothermal Clay - Horizon with hydrothermal origin | 86.2 |  |  |
| C0014B-4H-3, 17.0-37.0 cm | 27.640 | 27.840 | x |  |  | Clayey hydrothermal sand; Poorly sorted clay and sand comprising hydrothermally altered and mineralized material | 95.7 |  |  |
| C0014E-2H-6, 85.0-100.0 cm | 30.805 | 30.955 | x |  |  |  | 106.2 |  |  |
| C0014G-4H-5, 60.0-75.0 cm | 31.010 | 31.160 | x |  |  | Hydrothermal Clay - Horizon with hydrothermal origin | 106.8 |  |  |
| C0014B-4H-6, 93.0-103.0 cm | 31.470 | 31.570 | x |  |  | Hydrothermal Clay - Horizon with hydrothermal origin | 108.4 |  |  |
| C0014E-2H-7, 65.0-80.0 cm | 32.020 | 32.170 | x |  |  |  | 110.2 |  |  |
| C0014E-2H-8, 55.0-70.0 cm | 33.330 | 33.480 | x |  |  |  | 114.5 |  |  |
| C0014B-4H-8, 100.0-115.0 cm^*^ | 34.340 | 34.490 |  | 2445 |  | Hydrothermal Clay - Horizon with hydrothermal origin | 117.8 |  | 4633453.3 |
| C0014G-5H-3, 11.0-26.0 cm | 38.145 | 38.295 |  | 2606 |  | Hydrothermal Clay - Horizon with hydrothermal origin | 130.4 |  | 4633465.3 |
| C0014B-5H-12, 49.0-59.0 cm | 41.075 | 41.175 |  | 1003 |  | Hydrothermal Clay - Horizon with hydrothermal origin | 140.0 |  | 4633454.3 |
| C0014B-5H-15, 65.0-80.0 cm^*^ | 44.510 | 44.660 |  | 1585 |  | Hydrothermal Gravel - Matrix Supported | 151.4 | 150 | 4633455.3 |
| C0014G-21H-3, 0.0-15.0 cm | 99.105 | 99.255 | x |  |  | Hydrothermal Gravel - Matrix Supported | 331.5 |  |  |
| C0014G-24T-2, 39.0-54.0 cm | 110.090 | 110.240 | x |  |  | Hydrothermal Gravel - Matrix Supported | 367.8 |  |  |
| Extraction Blank - 28 PCR cycles |  |  |  | 16 |  |  |  |  | 4633450.3 |
| Extraction Blank - 35 PCR cycles |  |  |  | 405 |  |  |  |  | 4633451.3 |

^*^34 PCR cycles were used

**
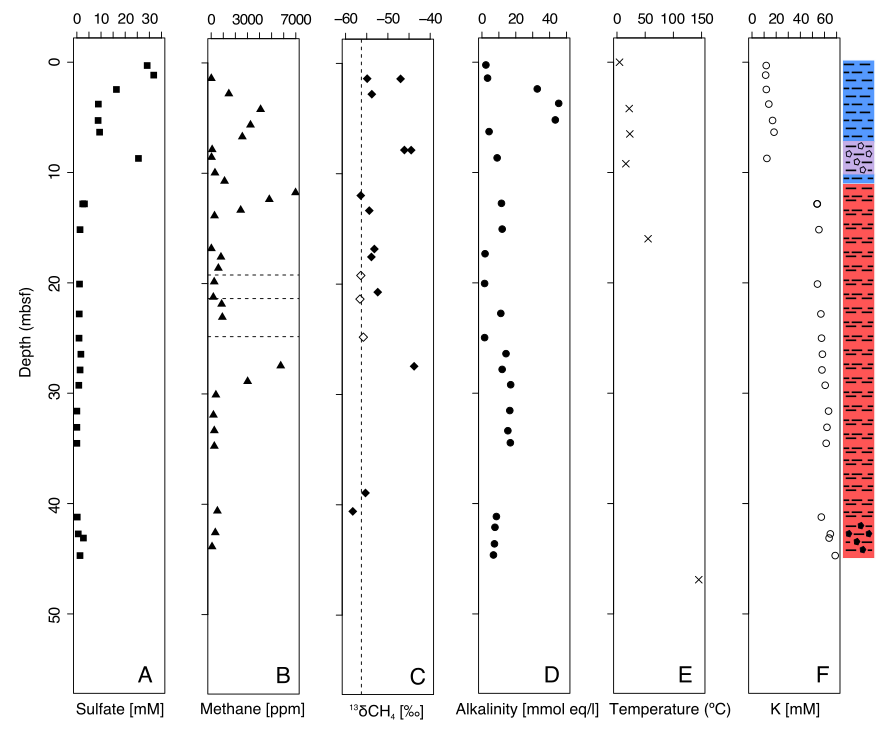
**

**Figure S2** Geochemical profiles with depth of IODP Site C0014 core. (A) Sulfate concentrations from Site C0014B reported in mM. (B) Methane concentrations from headspace gas samples of Site C0014B reported in ppm. The dashed lines represent the depths of collected safety gas samples (noticeable degassing on core cutting deck). (C) δ^13^CH_4_ measurements from Site C0014 (Holes B and D) samples reported in ‰ VPDB. The open diamonds represent the values of the safety-gas samples. The dashed vertical line is the average of the safety gas values. (D) Total alkalinity reported in mmol(eq)/l. (E) Temperature measurements in °C. (F) Potassium concentrations reported in mM. Abrupt change in K reflects the change in clay lithologies with depth. The lithologic representation is a modification from Takai *et al.* 2011. The first blue unit represents dark grayish brown silty clay. The purple unit represents pumiceous gravel/grit with dark grayish brown clay matrix. The first red unit represents a pale gray, heavily undurated hydrothermally altered clay. The deepest red unit represents a pale gray, heavily undurated hydrothermally altered clay with indurated mud clasts present.

**Table S2** List of all IODP Expedition 331 samples plotted in Figure S2(C) and their corresponding depth and δ^13^CH_4_ measurements. The depths are the averages of the Top and Bottom Depths.

**Supplemental Discussion of Methane Data**

Figure S2 shows several shipboard porewater chemistry measurements. The data shown in Figure S2(C) are the carbon isotopic measurements of methane samples collected from Site C0014 Holes B and D for land-based analyses. The three open diamonds represent safety gas samples, or samples in which methane and sulfide were noticeably degassing on the core cutting deck, implying extremely high concentrations of methane. Thus, it should be noted that the methane concentration measurements in Figure S2(B) at those depths are likely not accurate, as it was necessary for the core to sit on deck to degas. These depths are indicated in Figure S2(B) by horizontal dashed lines. The average of these three void gas measurements (-56.27 ‰), also considered to represent the source gas in this study, is represented by the vertical dashed line in Figure S2(C).

**Identification of External or Background DNA**

Due to the low concentrations of DNA of most sediment samples, a negative control carried through the extraction process was sequenced to account for any background DNA from the extraction kits. To account for any signal from the extraction kit in all samples, classification of reads was examined at the “fully expanded” taxonomic depth from the SILVA pipeline output, and all lineages present at the “order” level in the extraction blank in any amount were flagged and in all samples. Similarly, to account for external contamination from drilling processes, taxonomic “orders” identified from the seawater gel 16S rRNA clone analysis in Yanagawa *et al.*, 2013 were flagged if they 1) represented 5% or more of clones from their contamination analysis (includes data from holes B, E, and G), or 2) appeared in more than one hole from IODP Expedition 331 Site C0014.

**Taxonomic Classification Discrepancies**

The recent emergence of Thaumarchaeota, the deeply branching phylum within the Archaea, has spawned some archaeal classification disparities within 16S rRNA databases, namely, the SILVA SSU and the RefSSU databases. While most non-Euryarchaeota sequences were classified under the Thaumarchaeota phylum in the 454 dataset, the Illumina dataset referenced them as Crenarchaeota. Additionally, the Miscellaneous Crenarchaeotic Group has since been renamed as a phylum “Bathyarchaeota” instead of within the Thaumarchaeota. For the purposes of this study, we have grouped these taxa into the “Thaum- and Bathyarchaeota.”

**Supplemental Discussion of Amplicon Data**

Select samples were amplified using an archaeal primer set targeting the V6 hypervariable region and sequenced with Illumina technology (Table S1 and Figure S3). In order to demonstrate changes and trends in microbial diversity throughout the sediment column, Figure S3 shows archaeal sequences resolved to a deeper taxonomic level using two sequencing efforts. Between both datasets, there is good correspondence with respect to the observed proportion of Bathyarchaeota increasing with depth. Members of the highly diverse Bathyarchaeota are globally distributed in various marine and continental environments and are likely heterotrophic, using organic carbon derived from degradation of recalcitrant, fossil organic matter (Biddle et al., 2006; Kubo et al., 2012). Since uncultured representatives of the Bathyarchaeota defined only by 16S rRNA sequences are distinct from cultured Crenarchaeota, their ecological role in the subsurface is unclear (Inagaki et al., 2003; Kubo et al., 2012). Studies indicate that the MCG community is not active in methane or sulfur cycling (Biddle et al., 2006; Kubo et al., 2012), which agrees with the observed tradeoff in relative abundances between Bathyarchaeota and Methanomicrobia.

In both datasets, orders within Methanomicrobia are common throughout, with high abundances of ANME-1 at 0.305 mbsf and 15.295 mbsf. However, ANME-1 is overrepresented in the 454 sequencing results (Figure S3(A)) relative to those of Illumina Sequencing (Figure S3(B)), while it appears that the Illumina results have enhanced discrimination between ANME-1 and Methanosarcinales. Anaerobic methanotrophic archaea (ANME) are members of a microbial consortium involved in the anaerobic oxidation of methane in anoxic marine sediments (Boetius et al., 2000). The high relative abundance of ANME-1 represented in the 15.295 mbsf (~55°C) horizon indicates a potential methane oxidizing niche in the thermophilic regime. Although the magnitudes of ANME-1 relative abundances in Figure S2 are different between the two datasets, their consistent presence throughout the sediment profile suggests that methanotrophy is an important process in this hydrothermal environment.

The 454 dataset (Figure S3(A)) shows an overall decreasing trend in the Halobacteria and Methanomicrobia (*e.g.* Deep Hydrothermal Vent Euryarchaeotic Group 6 (DHVEG-6) and ANME-1, respectively) through 10.245 mbsf, where neither taxonomic class appears in the 12.87 or 12.99 mbsf horizons. The apparent tradeoff between the Halobacteria and Methanomicrobia classes and the Bathyarchaeota through the top 10.245 mbsf suggest that the Bathyarchaeota is less impacted by the increasingly temperature. Beginning at the 8.845 mbsf horizon, the Terrestrial Hot Spring Crenarchaeotic Group (THSCG) increases in relative abundance. At the 12.87 mbsf horizon, Archaea represent the majority of indigenous sequences (Figure 1), where the THSCG represent ~80% of archaeal sequences.

Interestingly, the taxa in the IODP Expedition 331 Site C0014 sediments are different than those from the surface sediments of IODP Expedition 331 Site C0015 (Figure S3(A)). The only commonality between the two sites is the presence of DHVEG-6. Unlike Site C0014, the upslope inactive Site C0015 shows virtually no taxa from Methanomicrobia. Approximately one-third of the archaeal sequences are represented by an uncultured Thermoplasmata, F2apm1A36, and nearly half of the archaeal sequences are represented by Marine Group I. Marine Group I has been found in surface layers of oxidized, organic-poor marine sediments (Teske, 2006; Teske and Sørensen, 2008) and seawater as prokaryotic picoplankton (DeLong et al., 1994; Teske and Sørensen, 2008). Additionally, culturing efforts have determined that Marine Group I represents aerobic, chemolithoautotrophic, nitrifying archaea that oxidize ammonia to nitrite (Könneke et al., 2005; Teske and Sørensen, 2008). Site C0015 exhibits an abundant occurrence of very permeable layers of pumice and volcaniclastic sediments, which has yielded porewater geochemistry profiles that are indistinguishable from seawater, suggesting recharge of seawater into the sediments (Takai et al., 2011). The presence of Marine Group I and evidence for a locally oxic surface layer suggests that surface processes are different between Sites C0014 and C0015, which ultimately shape the microbial community.

While the Illumina dataset (Figure S3(B)) differs in relative abundances among taxa compared with our primary sequence data from the V6-V9 amplicons (Figure S3(A)) in other represented taxa, the additional sequencing effort did fortunately enhance discrimination in the Thermoplasmata class (*i.e.* South African Gold Mine Group (SAGMEG), Thermoplasmatales, and the Terrestrial Miscellaneous Group). The results demonstrate the need for caution when working with taxonomic datasets from primer-amplified environmental DNA. Although an archaeal-specific primerset was used to complement the primary dataset, many archaeal lineages contain numerous mismatches compared with internal PCR primers and may be underrepresented (*e.g.* DHVEG-6, MG-I, and Ancient Archaeal Group) (Teske and Sørensen, 2008). Our work here aimed at effectively resolving key taxonomic groups in the heated hydrothermal subsurface of the Iheya North Field through the use of two different primersets. In spite of some variations between the two amplicon datasets, the subsurface sediments at IODP 331 Site C0014 in the Iheya North Field overall exhibit coherent and dramatic shifts in community with depth as temperature and hydrothermal influence increases.

Although the data in Figure S3(B) were analyzed via the VAMPS pipeline, we also analyzed the Illumina sequencing data with the SilvaNGS 1.1 pipeline (see methods). The results are plotted in Figure S4 and do not show any significant discriminations between the relative abundances of certain taxa, with the exception of better resolution within the Thermoplasmatales (purple and red shaded regions). Therefore, we assume that differences in taxonomic abundances are the result of biases in using different primers, rather than different pipelines and databases.


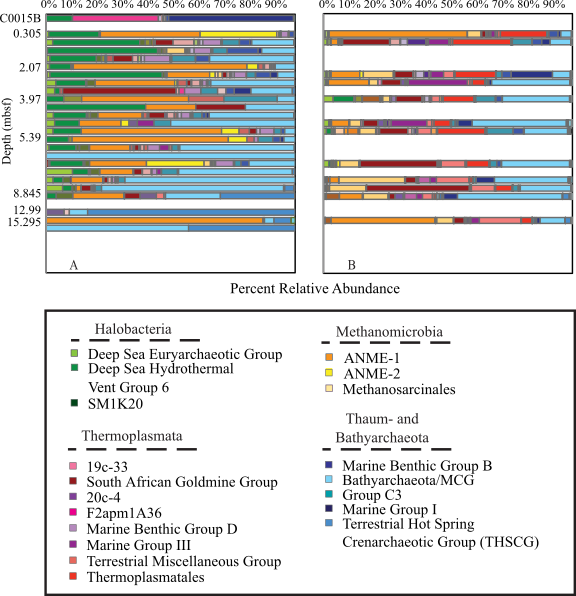


**Figure S3** Relative sequence abundances of archaeal 16S rRNA amplicons interpreted at the class level from 454 and Illumina sequencing efforts. Sample horizons are listed by increasing depth below seafloor. Site C0015, 600 m northwest and upslope of the hydrothermal vent, (shown separately as the topmost sample) showed no current hydrothermal activity and is being compared to represent non-hydrothermal conditions within the Iheya North Field. (A) Taxonomic dataset from 454 sequencing, using V6-V9 universal primers. Sequencing data were classified through the SILVA NGS pipeline. (B) Taxonomic dataset from Illumina sequencing, using V6 archaeal primers. Sequencing data were classified through the VAMPS pipeline.

**Figure S4** Relative sequencing abundance of Illumina sequencing dataset that was analyzed through the SILVA NGS pipeline

**Figure S5** The three horizons 6.74, 12.87, and 16.14 mbsf amplified Eukaryotic sequences (600, 58, and 11 total sequences respectively). Shown here are the relative proportions of those sequences that classified within Eukaryota. Note that C0014D-2-6 and C0014G-2-5 had significantly lower overall sequence yield. Both Basidiomycota and Ascomycota are fungal taxa, while Mollusca is an animal taxon.

References

Biddle, J.F., Lipp, J.S., Lever, M. a, Lloyd, K.G., Sørensen, K.B., Anderson, R., et al. (2006) Heterotrophic Archaea dominate sedimentary subsurface ecosystems off Peru. *Proc. Natl. Acad. Sci. U. S. A.* **103**: 3846–3851.

Boetius, A., Ravenschlag, K., Schubert, C.J., Dirk, R., Friedrich, W., Gieseke, A., et al. (2000) A marine microbial consortium apparently mediating anaerobic oxidation of methane. *Nature* **407**: 623–626.

DeLong, E.F., Wu, K.Y., Prezelin, B.B., and Jovine, R.V.M. (1994) High abundance of Archaea in Antarctic marine picoplankton. *Nature* **370**: 695–697.

Inagaki, F., Suzuki, M., Takai, K., Oida, H., Sakamoto, T., Aoki, K., et al. (2003) Microbial Communities Associated with Geological Horizons in Coastal Subseafloor Sediments from the Sea of Okhotsk. *Appl. Environ. Microbiol.* **69**: 7224–7235.

Könneke, M., Bernhard, A.E., de la Torre, J.R., Walker, C.B., Waterbury, J.B., and Stahl, D.A. (2005) Isolation of an autotrophic ammonia-oxidizing marine archaeon. *Nature* **437**: 543–546.

Kubo, K., Lloyd, K.G., F Biddle, J., Amann, R., Teske, A., and Knittel, K. (2012) Archaea of the Miscellaneous Crenarchaeotal Group are abundant, diverse and widespread in marine sediments. *ISME J.* **6**: 1949–1965.

Takai, K., Mottl, M.J., Nielsen, S.H., and The Expedition 331 Scientists (2011) Deep Hot Biosphere. *Proc. Integr. Ocean Drill. Progr.* **331.**

Teske, A. and Sørensen, K.B. (2008) Uncultured archaea in deep marine subsurface sediments: have we caught them all? *ISME J.* **2**: 3–18.

Teske, A.P. (2006) Microbial Communities of Deep Marine Subsurface Sediments: Molecular and Cultivation Surveys. *Geomicrobiol. J.* **23**: 357–368.

Yanagawa, K., Nunoura, T., McAllister, S.M., Hirai, M., Breuker, A., Brandt, L., et al. (2013) The first microbiological contamination assessment by deep-sea drilling and coring by the D/V Chikyu at the Iheya North hydrothermal field in the Mid-Okinawa Trough (IODP Expedition 331). *Front. Microbiol.* **4**: 1–10.
